# The Cytochrome P450-Mediated Metabolism Alternation of Four Effective Lignans From *Schisandra chinensis* in Carbon Tetrachloride-Intoxicated Rats and Patients With Advanced Hepatocellular Carcinoma

**DOI:** 10.3389/fphar.2018.00229

**Published:** 2018-03-14

**Authors:** Rongrong Wu, Zhiyong Xiao, Xiaorui Zhang, Feng Liu, Wenxia Zhou, Yongxiang Zhang

**Affiliations:** ^1^State Key Laboratory of Toxicology and Medical Countermeasures, Department of Neuroimmunopharmacology, Beijing Institute of Pharmacology and Toxicology, Beijing, China; ^2^Department of Pharmacy, 302 Hospital of People's Liberation Army, Beijing, China

**Keywords:** *Schisandra chinensis (SC)*, lignans, pharmacokinetics, liver injury, cytochrome P450(CYP450)

## Abstract

It is highly valuable to study the pharmacokinetics of herbal components under the pathological condition of liver dysfunction for safe and rational use of herbal medicines. In this study, the pharmacokinetic profiles of four effective lignans from *Schisandra chinensis (SC)*, schisandrin, schisantherin A, deoxyshisandrin and γ-schisandrin, were investigated in carbon tetrachloride (CCl_4_)-intoxicated rats. The metabolism of the four lignans was also studied using microsomes from patients with advanced hepatocellular carcinoma. *In situ* intestinal and hepatic perfusions were conducted to clarify the contributions from impairments of gut and liver on the pharmacokinetics of the four schisandra lignans in CCl_4_-intoxicated rats. The metabolism in rat and human liver microsomes and transport in Caco-2 monolayer cell model were studied to reveal the key factors for the *in vivo* disposition of the four lignans. When SC alcoholic extract was orally administrated to CCl_4_-intoxicated rat for a short term (4 days), the pharmacokinetics of four active SC lignans was significantly changed while its hepatotherapeutic effect was not obviously observed. The plasma concentrations of the four schisandra lignans were dramatically elevated compared with the control. The Cmax, AUC and MRT were all increased or prolonged significantly while parameter CLz/F was obviously reduced in rat pretreated with CCl_4_. In hepatic perfusion study and liver microsomes incubation, it was found that the hepatic metabolism of the four lignans was markedly decreased mainly due to the activity reduction of multiple CYP450 isoenzymes involved the metabolism, which, eventually, might lead to the alternation of their pharmacokinetic profiles in CCl_4_-intoxicated rats or patients with advanced hepatocellular carcinoma. The pharmacokinetic studies of SC components in pathological situation of liver dysfunction are expected to provide useful data for rational and safe application of SC preparations in clinic or further pharmacological and toxicological research.

## Introduction

Investigations revealed that the use of herbal medicines, usually as the complementary and alternative treatment, increased dramatically and became popular around the world (Ramos-Remus and Raut, 2008; Harris et al., 2012; Putthapiban et al., 2017). Meanwhile, the safety of herbal medicine and herbal-drug interactions have been highlighted and drawn extensive attention from the medical community (Brazier and Levine, 2003; Samuels et al., 2017; Singh and Zhao, 2017).

Liver is the most important organ for *in vivo* metabolism and biotransformation of xenobiotics irrespective of their origins. The pharmacokinetic profiles of drugs could be altered by the dysfunctions of liver in pathological conditions, which exerts a negative effect on drug safety and therapy (Tamura et al., 2004). Therefore, it is highly valuable to conduct the pharmacokinetic study of herbal components under the pathological condition of liver dysfunction, which could provide reliable and referential data for safe and rational clinical application of herbal drugs (McLean and Morgan, 1991; Yokogawa et al., 2004).

*Schisandra chinensis (SC)*, subjected to Magnoliaceae, comes from the dry mature fruit of *S. chinensis* (Turcz.) Baill or S. Sphenanchera Rehd. etWils. It has been used widely as a restorative or tonic in asthenic diseases in Asia and as a common constituent in many prescriptions in Traditional Chinese Medicines (TCM). Moreover, Wuweizi tablet (a preparation of an ethanol extract of SC) has been officially applied in clinic for treatment of liver diseases. Many pharmacological studies revealed that SC and schisandra lignans, the major effective components, showed various beneficial biological activities including hepatoprotection against viral and various hepatotoxins, tranquilization, hypnogenesis, anticonvulsive and neuro-protective effects, and so on (Lu and Liu, 1992; Fujihashi et al., 1995; Zhu et al., 2016; Szopa et al., 2017). However, it was recently reported that schisandrin B and schisandrae fructus oil could elevate hepatic and serum triglyceride levels, heighten serum alanine aminotransferase (ALT) activity, and eventually induce hepatotoxicity (hypertriglyceridemia, hepatomegaly and liver damage) in mice (Zhang et al., 2014). SC extracts exhibited inhibitive or inductive effects on rat hepatic cytochrome P450 (CYP450) enzymes and caused herb-drug interaction mediated by CYP450s (Wang et al., 2014). The pharmacokinetics of the SC lignans was correlated well with CYP3A, ALT and AST (Xie et al., 2010). Based on these former results, the pharmacokinetics of SC ingredients under conditions of liver dysfunctions should be further studied concerning that SC ingredients were often applied for treatment of various liver diseases.

**Figure 1:**
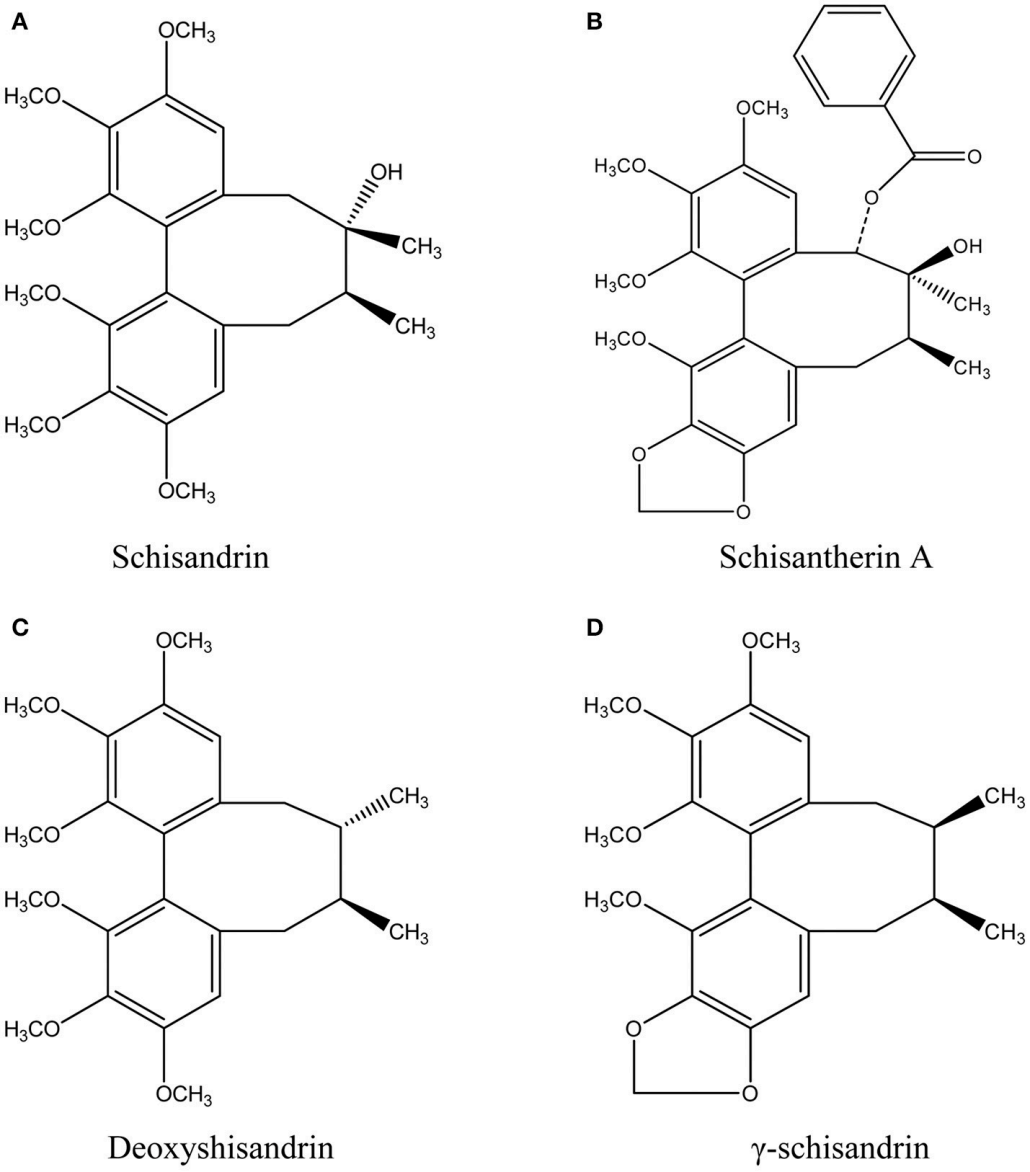
Chemical structures of schisandrin **(A)**, schisantherin A **(B)**, deoxyshisandrin **(C)**, and γ-schisandrin **(D)** from *Schisandra chinensis*.

In our present study, we selected four effective schisandra lignans with high abundance in SC alcoholic extract, schisandrin, schisantherin A, deoxyshisandrin and γ-schisandrin (Figure 1), to describe their pharmacokinetics in rat pretreated with carbon tetrachloride (CCl_4_), a classic hepatotoxin, using HPLC-MS method. Moreover, intestinal and hepatic perfusions were conducted to clarify the contributions from impairments of gut and liver on the pharmacokinetics of the four schisandra lignans in CCl_4_-intoxicated rats. The metabolism in rat and human liver microsomal incubations and transport in Caco-2 monolayer cell model were also studied to reveal the key factors for the *in vivo* disposition of the four lignans. The results of the study are expected to provide useful data for rational and safe application of SC preparations in clinic.

## Materials and methods

### Materials

Dried alcoholic extract of SC containing schisandrin (1.83%), schisantherin A (1.52%), deoxyshisandrin (2.41%) and γ-schisandrin (1.22%), was purchased from Nantong Sihai Plant Extracts Co., Ltd (Jiangsu, China). Schisandrin, schisantherin A, deoxyshisandrin, γ-schisandrin (>99% purity) and midazolam were purchased from the National Institute for the Control of Pharmaceutical and Biological Products (Beijing, China). Phenacetin, acetaminophen, dextromethorphan, dextrophan, diclofenac, 4-hydroxydiclofena, mephenytoin, 4-hydroxymephenytoin, chlorzoxazone, 6-hydroxychlorzoxazone, 1-hydroxymidazolam, ketoconazole, ticlopidine, sulfaphenazole, quinidine, furafylline, disulfiram were obtained from Sigma-Aldrich Corporation (MO, US). Bicyclol (>99% purity) was provided by Beijing Union Pharmaceutical Plant. CCl_4_ and glycerin were purchased from Beijing Chemical Reagents Company. Methanol and acetonitrile were HPLC grade (Fisher, USA), other reagents were analytical reagent grade. The pooled male human microsomes were purchased from BD corporation (MA, US). Ultrapure water was prepared using Milli-Q Reagent System (Millipore, MA, USA); AST, ALT, ALP, LDH, TB, BUN, and CRE kits were purchased from Nanjing Jiancheng Reagents Company.

### Animal treatment

Male Sprague-Dawley rats, weighing 180–220 g, were obtained from Beijing Vital River Experimental Animal Co., Ltd. The animals were quarantined for 1 week prior to study and maintained on a 12-h light/12-h dark cycle at 22 ± 1°C and at 50–55% relative humidity with free access to solid pellet diet and water *ad libitum* throughout the study. All rats were assigned randomly to three groups, including acute liver injury (ALI) rat, SC-treated ALI rat and the control, 5 in each group. The ALI and SC-treated ALI rats were intraperitoneal injected two doses of the mixture of CCl_4_ and olive oil (1:1, V/V) at 2 mL/kg in a 24 h time interval (Huang et al., 2001), while the control group was given the same volume of olive oil. Then, the SC-treated ALI rat was orally dosed with SC alcoholic extract suspended in 0.5% sodium carboxymethyl cellulose (CMC) at 1.5 g/kg/day for 4 days after CCl_4_ injection. The dosages were set according to the amounts of the four active lignans used previously for pharmacological studies and their contents in the extract (Chiu et al., 2002, 2007; Cheng et al., 2013). All animal protocols were approved by the Animal Care and Welfare Committee of the Academy of Military Medical Sciences. All animal procedures complied with the Guide for the Care and Use of Laboratory Animals issued by Beijing Association on Laboratory Animal Care (BALAC).

### Assays for biochemical indexes

Serum aspartate transaminase (AST), alanine transaminase (ALT), alkaline phosphatase (ALP), lactate dehydrogenase (LDH), total bilirubin, blood urea nitrogen (BUN), and creatinine (CRE) levels were determined with commercial kits (Nanjing Jiancheng Bioengineering Institute, China).

### Pharmacokinetic studies of four effective lignans in ALI rat

The pharmacokinetic profiles of four lignans were studied in ALI rat after multiple oral administrations of SC alcoholic extract at dose of 1.5 g/kg/day. After the ALI rat was established, SC alcoholic extract suspended in 0.5% sodium carboxymethyl cellulose was given to ALI rats and the control *via* oral gavage once a day for 4 consecutive days. Before the last dosing, rats were fasted for 12 h while water was taken freely. Blood samples were collected at 0, 0.25, 0.5, 1, 2, 3, 4, 6, 8, 12, 24, 36, and 48 h after the last dosing. A cannula was surgically placed into the jugular vein for collection of blood samples (about 0.1 mL) and blood loss compensation using sterile isotonic saline in the pharmacokinetic study. Plasma was separated by centrifugation and stored at −20°C until analysis. Plasma concentrations of the four lignans were quantified using HPLC-MS analysis developed according to the reported method (Wang et al., 2008).

### *In situ* intestinal perfusion of four effective lignans

The surgical procedures of intestinal perfusion were conducted according to those published reports (Savina et al., 1981). Hank's balanced salt solution (HBSS, pH 7.4) with four schisandra lignans (50 μM) at 37°C was infused into the jejunum segment at rate of 0.3 mL/min. Phenol Red (50 μM) was added to serve as a nonabsorbable marker for measuring water flux. Blood samples from mesenteric vein and perfusate were collected at 5-min interval. The plasma separated by centrifugation and perfusate was stored at −20°C until HPLC-MS analysis.

### *In situ* hepatic perfusion studies of four effective lignans

*In situ* liver perfusion was performed similarly as the method described previously (Uraki et al., 2016). Rats were cannulated in the bile duct, hepatic portal vein, and inferior vena cava. The perfusate consisted of Krebs–Hensleit buffer (pH 7.4) containing sodium taurocholate (4.75 mg/L), 1% bovine serum albumin, and glucose (1.2 g/L) was oxygenated using carbogen, 95% O_2_/5% CO_2_. The perfusate at 37°C was infused into the portal vein at a flow rate of 30 mL/min in a recirculatory manner. After 10-min equilibration, four schisandra lignans were added to the reservoir (final concentration of 10 μM). Perfusate (0.5 mL) and bile samples were collected at 0, 10, 20, 30, 40, 50, and 60 min after the addition of lignans. The same volume of blank perfusion buffer was immediately added to the reservoir after collection of the perfusate samples. After hepatic perfusion, rat was sacrificed and liver was removed, blotted dry, and weighed.

### Measurement of cytochrome P450 activities with probe substrates

Liver microsomes of ALI rats and patient with advanced hepatocellular carcinoma (AHC) were prepared by differential centrifugation as described previously (Eriksson, 1978). The study protocol preparing liver microsomes from patients was approved by the Ethics Committee of 302 Hospital of People's Liberation Army. The pellet was resuspended in homogenization medium and stored at −80°C until use. The CYP450 isozymes activities were determined by probe substrate method reported previously (He et al., 2007; Li et al., 2007). The activities of CYP isoforms were indicated by the formation rates of the specific metabolites of six probe substrates (phenacetin for rat and human CYP1A2, diclofenac for rat CYP2C6 and human CYP2C9, S-mephenytoin for rat CYP2C11 and human CYP2C19, dextromethorphan for rat CYP2D2 and human CYP2D6, chlorzoxazone for rat and human CYP2E1, and midazolam for rat CYP3A1/2 and human CYP3A4/5) incubated in rat and human liver microsomes. All metabolites were determined using validated LC-MS/MS methods.

### Metabolism of four schisandra lignans in rat or human liver microsomes incubation

The metabolism of four schisandra lignans was investigated in liver microsomes prepared from CCl_4_-intoxicated rat or AHC patient. The incubation system (200 μL, ≤ 1% of organic solvent) included rat (final protein concentration: 1 mg/mL) or human (final protein concentration: 0.5 mg/mL) liver microsomes, NADPH (1.2 mM), Tris-Hcl buffer (50 mM, pH = 7.4) and four lignans (final concentration was 5 μM). After 5 min preincubation in water bath at 37°C, NADPH was added to start the reaction, after a series of given time incubation (0, 5, 10, 20, 30, 45, 60 min), 400 μL ice acetonitrile was added to stop the reaction followed by centrifugation (14,000 rpm × 5 min). The four lignans in the supernate were analyzed by the similar HPLC-MS method in the pharmacokinetic study.

### Identification of CYP450 isozymes involved in the metabolism of four schisandra lignans in rat or human liver microsomes (Rendic and Di Carlo, 1997)

The CYP450s involved in the metabolism of schisandrin, schisantherin A, deoxyshisandrin and γ-schisandrin in rat or human liver microsomes were confirmed by comparison of the metabolism ratio before and after the addition of selective inhibitors to CYP450 isozymes. The incubation system includes rat liver microsomes (1 mg/mL) or human liver microsomes (0.5 mg/mL), NADPH (1.2 mM), Tris-Hcl buffer (50 mM, pH = 7.4), four lignans (final concentration was 5 μM), and CYP450 isozymes inhibitors (CYP3A/ ketoconazole 100 μM; CYP2C11/ticlopidine 50 μM; CYP2C6/ sulfaphenazole 50 μM; CYP2D2/ quinidine 50 μM; CYP1A2/ furafylline 50 μM; CYP2E1/disulfiram 250 μM), while the organic solvent volume was not more than 1% of total volume (500 μL). After 5 min preincubation in water bath at 37°C, NADPH was added to start the reaction (furafylline and disulfiram whose metabolites actually inhibited the activities of CYP450s were preincubated 15 min in the present of NADPH before substrate addition, other steps were similar), after a given time incubation (15 min for schisandrin, 40 min for schisantherin A, deoxyshisandrin and γ-schisandrin, the time was set by a pre-experiment), 500 μL ice acetonitrile was added to stop the reaction followed by centrifugation. The supernate was injected to HPLC-MS for analysis of the four lignans.

### Transport studies in Caco-2 cell monolayer model

Caco-2 cell (passage 38) was used in the transport experiments. Cells were seeded on polycarbonate filters (12 mm diameter, pore size 0.4 mm) at 1 × 10^5^/cm^2^. The cell monolayer was formed 21 days post seeding when the transepithelial electrical resistance (TEER) was above 1,000 Ω• cm^2^. Three concentration levels (5, 10, and 50 μM) were set for the four schisandra lignans in the study. After preincubation for 1 h at 37°C, the solution of the four lignans was respectively added to the apical (0.4 mL) or basolateral (0.6 mL) chamber for absorptive (AP → BL) and secretive (BL → AP) permeability test. At the specified timepoints (0.5, 1, 1.5 and 2 h), 50 μL samples (*n* = 3) were withdrawn from receiver chamber, and meanwhile, 50 μL fresh buffer was added. The four lignans were determined by HPLC-MS method.

### Data analysis

The permeability of four lignans across the rat intestine was calculated based on the disappearance of parent drug from the lumen (P_lumen_) as well as the appearance of the drug in blood (P_blood_) using the following equations (Singhal et al., 1998):

(1) $${\text{P}}_{\text{lumen}}\;=\;-\frac{Q}{2\pi \text{rl}}Ln\frac{C_{out}}{C_{in}}$$

(1) $${\text{P}}_{\text{blood}}\;=\;\frac{\Delta M_{B}/\Delta t}{2\pi rl\{C\}}$$

where *r* is the radius of the perfused intestinal lumen; *l* is the length of the segment; Q is the flow rate of perfusate; C_in_ is the concentration of drug at the start of perfusion; and C_out_ is the steady-state concentration of drug exiting the lumen. ΔM_B_/Δt is the rate of drug appearance in mesenteric blood; and {C} is the logarithmic mean concentration of drug in the lumen.

The apparent permeability coefficient (P_app_) in the Caco-2 cell was calculated by the formula P_app_ = (ΔQ/Δt)/AC_0_, where ΔQ/Δt is the transport rate (nmol/s), which remained approximately constant in time, A is the well membrane area (0.6 cm^2^), and C_0_ is the initial concentration of the four lignans (μM). The efflux rate (ER) was calculated by the formula ER = P_app (BL → AP)_/P_app(AP → BL)._

### Pharmacokinetics analysis

Data fitting and pharmacokinetic parameter calculations were carried out using the Winnolin software. Non-compartmental analysis was used to calculate the pharmacokinetic parameters. Parameters, such as AUC and MRT, were calculated using statistical moment method.

### Statistical analysis

Statistically significant difference between two groups was evaluated by Student's *t*-test. Non-parametric tests was used to analyze Tmax or the data failed in homogeneity of variance test. The prior level of significance was set at *P* < 0.05. All results are expressed as mean ± standard deviation (*SD*).

## Results

### The evaluation of liver and renal functions in rat intoxicated with CCl_4_

The functions of liver and kidney in rat intoxicated with CCl_4_ were evaluated by determining the related biochemical indexes (Table 1). The results showed that the values of liver index, serum ALT and AST, and BUN was increased by 40, 720, 30, and 30% in ALI rat intoxicated with CCl_4_, while the content of liver microsomal CYP450 enzyme was decreased by 56% compared with control rats. In addition, it was found that the 4-day administration of SC alcoholic extract (1.5 g/kg/day) did not show positive effect on the liver dysfunction in ALI rats indicated by elevated serum ALT and AST or decreased CYP450 content.

**Table 1:** Indexes of liver and renal functions in acute liver injury (ALI) rats intoxicated by carbon tetrachloride.

**Biochemical indexes**	**Control**	**CCl_4_-intoxicated rats**	**SC-treated ALI rats**
Liver index (g/kg)	26 ± 3	38 ± 5^*^	35 ± 2^*^
Liver P450 (nmol/mg)	0.59 ± 0.09	0.27 ± 0.12^**^	0.31 ± 0.03^**^
ALT (IU/L)	40 ± 13	329 ± 49^**^	333 ± 26^**^
AST (IU/L)	195 ± 5	268 ± 49^*^	280 ± 9^*^
ALP (IU/L)	6.36 ± 0.04	8.15 ± 0.09^*^	7.72 ± 0.19^*^
LDH (IU/L)	567 ± 154	605 ± 168	551 ± 82
TB (mg/dL)	0.12 ± 0.07	0.09 ± 0.02	0.13 ± 0.02
BUN (mg/dL)	14.0 ± 0.7	18.3 ± 1.2^*^	17.3 ± 0.7^*^
CRE (mg/dL)	0.9 ± 0.1	0.8 ± 0.1	0.8 ± 0.1

*Data are expressed as the mean ± standard deviation (SD), n = 6. CCl_4_, carbon tetrachloride; SC, Schisandra chinensis; ALI, acute liver injury; AST, serum asparate transaminase; ALT, alanine transaminase; ALP, alkaline phosphatase; LDH, lactate dehydrogenase; BUN, blood urea nitrogen; CRE, creatinine. Statistical analysis was performed using Student's t-test between control and CCl_4_-intoxicated or SC-treated ALI rats*,

* *P < 0.05*,

** *P < 0.01*.

These data suggested that the CCl_4_ injection caused significant liver injury and little alteration in renal function, which is mostly consistent with the previous study. Short-term administration of SC alcoholic extract may not exhibit hepatotherapeutic effect.

### Pharmacokinetics of the four lignans in CCl_4_-intoxicated rats after orally dosing of SC alcoholic extract

**Figure 2:**
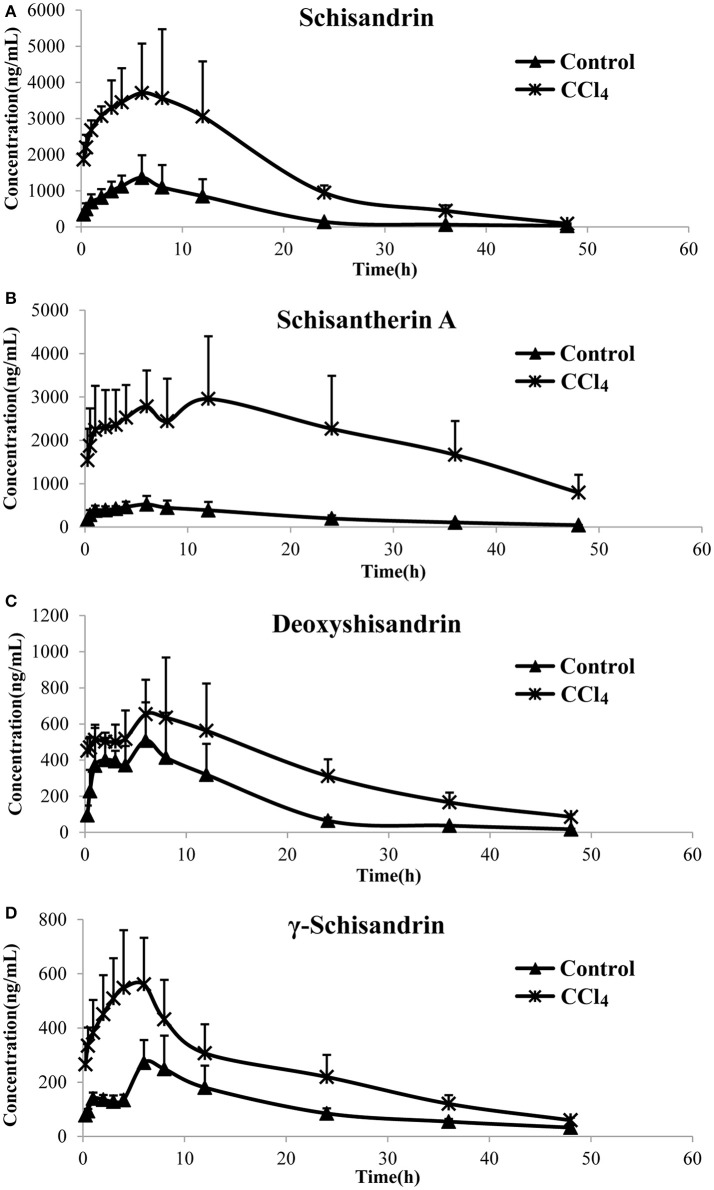
Plasma concentration–time curves of the four lignans in carbon tetrachloride (CCl_4_)-intoxicated rats after oral administration of alcoholic extract of *Schisandra chinensis* (1.5 g/kg, *n* = 5). Control, group of normal rats; CCl_4_, group of CCl_4_-intoxicated rats; **(A)** schisandrin; **(B)** schisantherin A; **(C)** deoxyshisandrin; **(D)** γ-schisandrin.

The pharmacokinetic profiles of the four lignans were investigated in CCl_4_ intoxicated rat after orally multiple dosing of SC alcoholic extract at 1.5 g/kg. The results showed that most of the pharmacokinetic parameters of the four lignans including Cmax, AUC and MRT were significantly increased in CCl_4_-treated group, compared with control rats that received vehicle alone (Figure 2, Table 2). In addition, the systemic clearance (CLz/F) in CCl_4_ intoxicated rats was reduced by about 83% for schisandrin, 89% for schisantherin A, 56% for deoxyshisandrin and 54% for γ-schisandrin, respectively. These results suggested that the *in vivo* disposition of the lignans was dramatically altered in liver-injured rats pretreated with CCl_4_.

**Table 2:** Pharmacokinetic parameters of four lignans in carbon tetrachloride -intoxicated rats after oral administration of alcoholic extract of *Schisandra chinensis* at 1.5 g/kg (*n* = 5).

**Compounds**	**Group**	**t_1/2_**	**C_max_**	**T_max_**	**AUC_(0−t)_**	**MRT_(0−t)_**	**CLz/F**
		**(h)**	**(mg/L)**	**(h)**	(mg/L^*^h)	**(h)**	**(L/h/kg)**
Schisandrin	Control	10.08 ± 4.12	1367.19 ± 616.91	6	19845.50 ± 7492.17	11.05 ± 0.97	1460.01 ± 363.74
	CCl_4_	7.74 ± 1.21	3984.58 ± 1530.91^**^	6.20 ± 2.05	74727.40 ± 26325.22^**^	12.82 ± 0.25^**^	396.38 ± 118.55^***^
Schisantherin A	Control	12.39 ± 2.12	568.88 ± 162.71	5.00 ± 1.41	11319.05 ± 3871.60	15.42 ± 0.29	2040.35 ± 542.59
	CCl_4_	17.65 ± 3.32^*^	3190.60 ± 1164.05^**^	9.60 ± 3.29	99613.98 ± 45576.10^**^	19.84 ± 1.18^***^	234.05 ± 115.74^***^
Deoxyshisandrin	Control	11.53 ± 2.08	563.77 ± 318.48	5.00 ± 2.24	7883.39 ± 2813.91	11.69 ± 0.97	4799.88 ± 1240.08
	CCl_4_	13.08 ± 1.78	774.61 ± 239.38	5.80 ± 2.86	16409.50 ± 5021.78^*^	16.39 ± 1.02^***^	2127.42 ± 459.69^**^
γ-Schisandrin	Control	15.41 ± 2.52	181.66 ± 102.83	6.40 ± 0.89	5223.03 ± 1431.07	16.52 ± 0.94	3256.49 ± 685.44
	CCl_4_	14.49 ± 5.05	627.84 ± 163.72^**^	4.20 ± 1.79	11565.78 ± 3092.54^**^	15.94 ± 1.24	1509.03 ± 354.86^**^

*Data in Figure 2 were used for calculation of pharmacokinetic parameters. CCl_4_, carbon tetrachloride. The results are shown as means ± SD (n = 5). Statistical analysis was performed using Student's t-test—compare between Control and intoxicated rats. Non-parametric tests was used to analyze Tmax or the data failed in homogeneity of variance test*.

* P < 0.05;

** P < 0.01;

*** *P < 0.001*.

### *In situ* intestinal and hepatic perfusion of four lignans

*In situ* intestinal and hepatic perfusion was conducted in our study to evaluate the effect of intestinal absorption and hepatic metabolism on the pharmacokinetic profiles of four schisandra lignans in CCl_4_-intoxicated rats.

As shown in Table 3, the remaining amounts of schisandrin and deoxyshisandrin in perfusate were both slightly increased about 10% in intestinal autoperfusion (60 min) of CCl_4_-treated rats, while those of schisantherin A and γ-schisandrin were not changed compared with the control. Besides, similar results were also found in the cumulative plasma amounts of schisandrin and deoxyshisandrin which raised about 91% and 63% in CCl_4_-intoxicated rats. The other parameters including cumulative intestinal amount, P_blood_ and P_lumen_ were not obviously altered in CCl_4_-treated rats.

**Table 3:** Determination of the four lignans in perfusate, intestinal tissue, and plasma after intestinal perfusion *in situ*.

**Compounds**	**Group**	**Perfusate**	**Intestine**	**Plasma**	**P_blood_**	**P_lumen_**
		**(Percent of the initial dose)**	**(Percent of the initial dose)**	**(Percent of the initial dose)**	**(× 10^−4^ cm/s)**	**(× 10^−4^ cm/s)**
Schisandrin	Control	86.16 ± 1.39	2.22 ± 0.96	0.44 ± 0.06	0.046 ± 0.008	2.490 ± 0.141
	CCl_4_	93.40 ± 1.05^**^	1.98 ± 0.13	0.84 ± 0.06^**^	0.063 ± 0.008	2.420 ± 0.788
Schisantherin A	Control	92.47 ± 1.06	1.65 ± 0.37	0.80 ± 0.07	0.047 ± 0.006	2.303 ± 0.163
	CCl_4_	92.85 ± 1.33	1.95 ± 0.36	0.82 ± 0.04	0.060 ± 0.016	2.047 ± 0.373
Deoxyshisandrin	Control	81.69 ± 1.77	1.69 ± 0.30	0.51 ± 0.03	0.053 ± 0.009	2.337 ± 0.211
	CCl_4_	91.77 ± 1.59^**^	1.79 ± 0.41	0.83 ± 0.06^**^	0.063 ± 0.018	2.270 ± 0.363
γ-Schisandrin	Control	92.57 ± 1.67	1.66 ± 0.27	0.84 ± 0.09	0.052 ± 0.009	2.543 ± 0.174
	CCl_4_	91.92 ± 1.69	1.87 ± 0.20	0.82 ± 0.06	0.066 ± 0.018	2.540 ± 0.423

*Data are expressed as the mean ± standard deviation (SD), n = 3. CCl_4_, carbon tetrachloride. Statistical analysis was performed using Student's t-test—compare between Control and CCl_4_-intoxicated rats*,

** *P < 0.01*.

In hepatic perfusion study, the results showed that the AUC_(0−1h)_ of four schisandra lignans in perfusate and their cumulative amounts in liver were significantly increased. The total amount of deoxyshisandrin in bile was reduced in CCl_4_-treated rats (Table 4). The above data demonstrated that the hepatic metabolism of the four lignans was markedly reduced in rats pretreated with CCl_4_.

**Table 4:** Determination of four lignans in perfusate, liver tissue, and bile after hepatic perfusion *in situ*.

**Compounds**	**Group**	AUC (mg/L ^*^h)	**Total amount of analyte in bile (μg)**	**Total amount of analyte in liver (μg)**
Schisandrin	Control	3.23 ± 0.11	1.15 ± 0.27	11.19 ± 1.18
	CCl_4_	3.86 ± 0.07^**^	1.06 ± 0.14	19.55 ± 2.07^**^
Schisantherin A	Control	4.08 ± 0.14	1.48 ± 0.23	12.36 ± 1.65
	CCl_4_	4.79 ± 0.03^***^	1.61 ± 0.34	17.15 ± 1.84^*^
Deoxyshisandrin	Control	3.09 ± 0.12	1.29 ± 0.30	9.71 ± 1.32
	CCl_4_	3.62 ± 0.10^**^	0.75 ± 0.07^*^	14.57 ± 2.41^*^
γ-Schisandrin	Control	3.19 ± 0.07	1.37 ± 0.14	11.10 ± 1.31
	CCl_4_	3.74 ± 0.07^***^	1.19 ± 0.28	14.25 ± 1.36^*^

*Data are expressed as the mean ± standard deviation (SD), n = 3. AUC, area under the curve; CCl_4_, carbon tetrachloride. Statistical analysis was performed using Student's t-test between Control and CCl_4_ –intoxicated rats*,

* *P < 0.05*,

** *P < 0.01*,

*** *P < 0.001*.

### Determination of liver microsomal CYP450 isozymes activities

**Figure 3:**
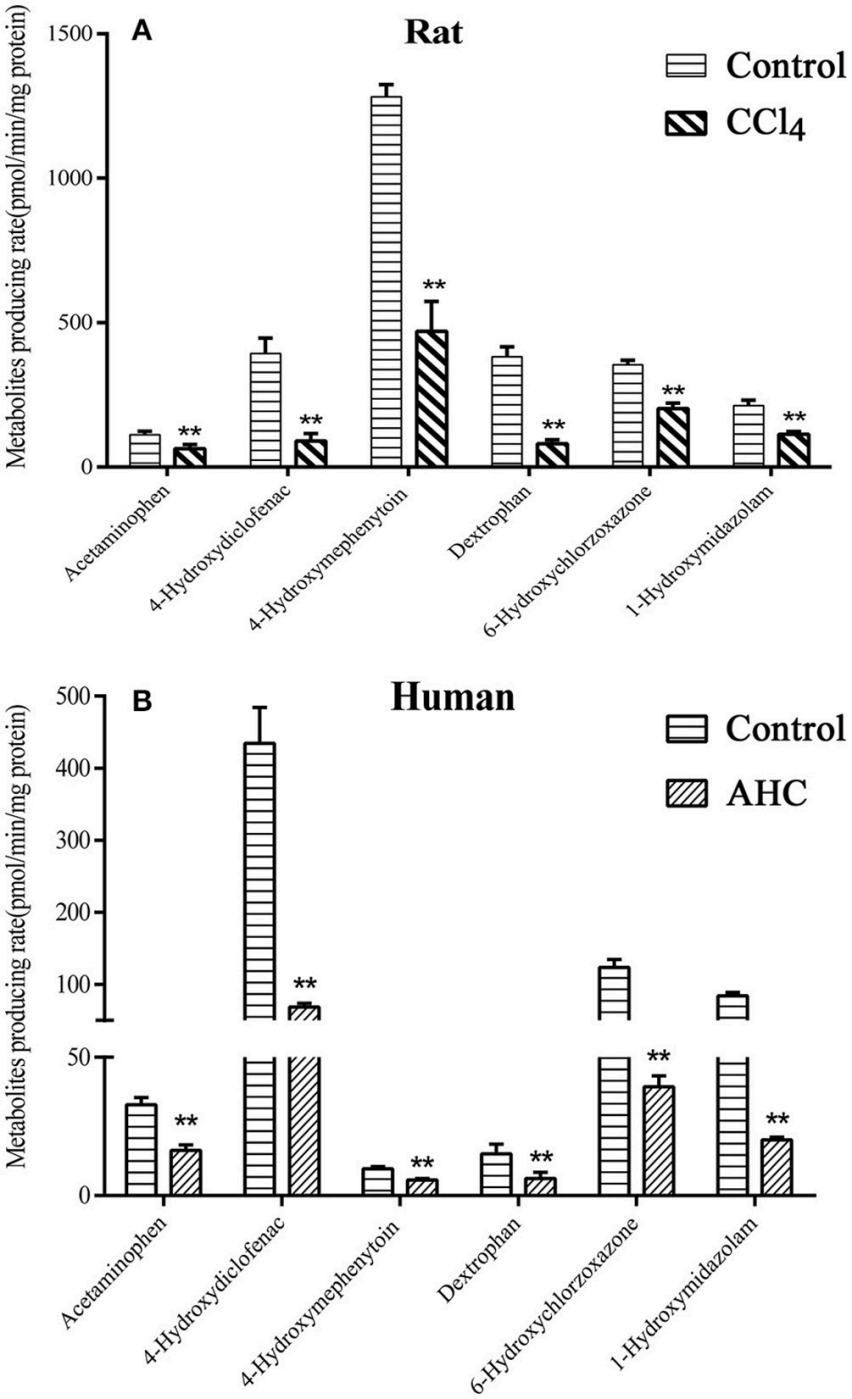
*In vitro* metabolites producing rate of CYP450s probe substrates in liver microsomal incubation of carbon tetrachloride (CCl_4_)-intoxicated rats **(A)** and patients **(B)** with advanced hepatocellular carcinoma (AHC). **(A)** Control, liver microsomes from normal rats; CCl_4_, liver microsomes from CCl_4_-intoxicated rats; **(B)** Control, pooled male human liver microsomes purchased from BD corporation; AHC, liver microsomes prepared from patients with advanced hepatocellular carcinoma. Acetaminophen-rat and human CYP1A2; 4-Hydroxydiclofenac-rat CYP2C6 and human CYP2C9; 4-Hydroxymephenytoin-rat CYP2C11 and human CYP2C19; Dextrophan-rat CYP2D2 and human CYP2D6; 6-Hydroxychlorzoxazone- rat and human CYP2E1; 1-Hydroxymidazolam- rat CYP3A1/2 and human CYP3A4/5. Statistical analysis was performed using Student's *t*-test, ^**^P < 0.01, vs. CCl_4_-intoxicated rat or AHC patient, *n* = 5.

The activities of six liver CYP450 isozymes in CCl_4_-intoxicated rat or patient with advanced hepatocellular carcinoma (AHC) were subsequently determined by method of probe substrates incubation in liver microsomes. The results, shown in Figure 3, demonstrated that the metabolic production rates of the six probe substrates in liver microsomes of CCl_4_-intoxicated rats were significantly decreased by 43% for acetaminophen (CYP1A2), 77% for 4-hydroxydiclofenac (CYP2C6), 63% for 4-hydroxymephenytoin (CYP2C11), 79% for dextrophan (CYP2D2), 43% for 6-hydroxychlorzoxazone (CYP2E1), and 46% for 1-hydroxymidazolam (CYP3A), respectively. Likewise, the activities of six liver CYP450s from AHC patient were reduced by about 50% for CYP1A2, 87% for CYP2C9, 80% for CYP2C19, 66% for CYP2D6, 68% for CYP2E1, and 76% for CYP3A4/5.

### Metabolism of four schisandra lignans in rat or human liver microsomes incubation

**Figure 4:**
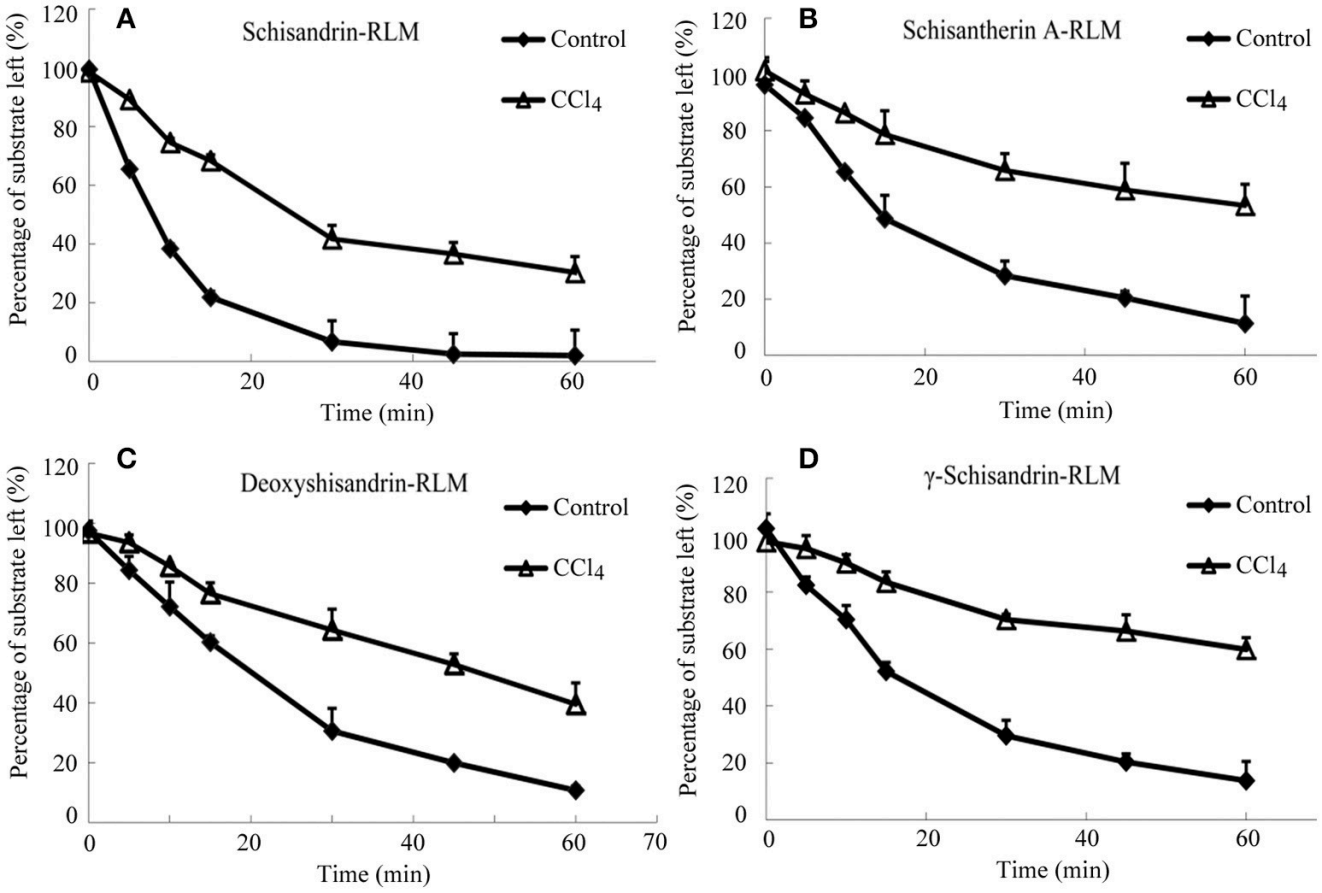
Substrate left-incubation time curve of schisandrin **(A)**, schisantherin A **(B)**, deoxyshisandrin **(C)**, and γ-schisandrin **(D)** in carbon tetrachloride (CCl_4_)-intoxicated rat liver microsomes (*n* = 3). Control, liver microsomes from normal rats; CCl_4_, liver microsomes from CCl_4_-intoxicated rats.

**Figure 5:**
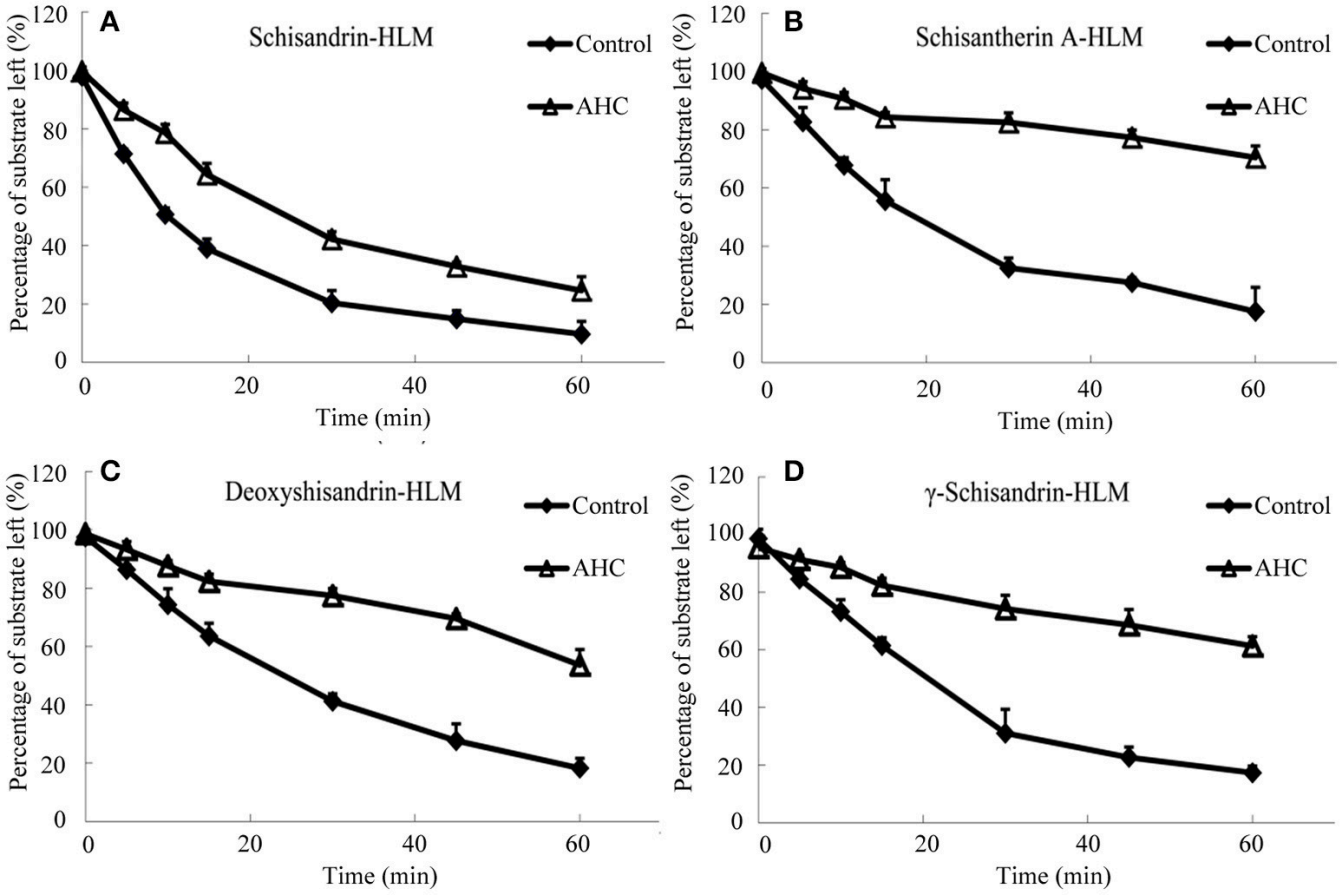
Substrate left-incubation time curve of schisandrin **(A)**, schisantherin A **(B)**, deoxyshisandrin **(C)**, and γ-schisandrin **(D)** in liver microsomes from patient with advanced hepatocellular carcinoma (AHC) (*n* = 3). Control, pooled male human liver microsomes purchased from BD corporation; AHC, liver microsomes prepared from patients with advanced hepatocellular carcinoma.

It was investigated the metabolism of four schisandra lignans *in vitro* using rat or human liver microsomes from CCl_4_ intoxicated rat or patient with advanced hepatocellular carcinoma (AHC). The metabolism of the four lignans was indicated by the percentage of the substrate reduced after incubation compared with the initial concentration. As shown in Figures 4, 5, the substrate left percentage of four lignans was 30.2–59.9% in CCl_4_-treated rat liver microsomes incubation, while that was 1.92–13.74% in control samples after incubation for 60 min. Likely, the substrate left percentage was 24.6–70.4% for four schisandra lignans in AHC patient liver microsomes incubation compared with 9.7–18.3% of four lignans left in control group. All these results indicated that the *in vitro* metabolism of four schisandra lignans was reduced in liver microsomes from CCl_4_-treated rat and AHC patient.

### Identification of CYP450 isozymes involved in the metabolism of four schisandra lignans in rat and human liver microsomes

The CYP450 isozymes involved in the metabolism of the four effective schisandra lignans were identified in rat and human liver microsomes (Tables 5, 6). In the rat liver microsomes incubation, it was found that multiple CYP450 isozymes participated in the metabolism of the four lignans (≥20% inhibition), i.e., CYP2C6/11, CYP2E1, and CYP3A for schisandrin, CYP1A2, CYP2C6/11, and CYP2E1 for schisantherin A, CYP2C11, CYP2D2, CYP2E1, and CYP3A for deoxyshisandrin, and CYP1A2, CYP2D2 and CYP2E1 for γ-schisandrin. Likewise, multiple CYP450s were found involved in the metabolism of the four compounds in human liver microsome incubation. These results proved CYP450s played a key role in the metabolism of four schisandra lignans.

**Table 5:** Effect of CYP450s inhibitors on four lignans metabolism in rat liver microsomes.

**Groups**	**CYP450s**	**Schisandrin**		**Schisantherin A**		**Deoxyshisandrin**		γ**-Schisandrin**	
		**Metabolism rate (nmol/min/mg)**	**Inhibition (%)**	**Metabolism rate (nmol/min/mg)**	**Inhibition (%)**	**Metabolism rate (nmol/min/mg)**	**Inhibition (%)**	**Metabolism rate (nmol/min/mg)**	**Inhibition (%)**
Control	–	233.3 ± 35.6	–	91.3 ± 11.2	–	87.5 ± 7.8	–	79.6 ± 6.5	–
Furafylline	CYP1A2	215.1 ± 26.5	7.8	48.0 ± 6.4	47.4	85.2 ± 7.6	2.6	35.5 ± 4.1	55.4
Ticlopidine	CYP2C11	184.8 ± 14.5	20.8	24.0 ± 1.4	73.7	20.4 ± 3.2	76.7	77.5 ± 5.6	2.6
Sulfaphenazole	CYP2C6	185.2 ± 17.6	20.6	48.9 ± 5.3	46.4	84.8 ± 10.6	3.1	70.6 ± 8.2	11.3
Quindine	CYP2D2	228.2 ± 30.5	2.2	88.4 ± 9.4	3.2	27.7 ± 3.4	68.4	62.4 ± 5.9	21.6
DETC	CYP2E1	133.9 ± 23.1	42.6	33.8 ± 3.1	63	23.4 ± 2.7	73.3	30.8 ± 4.2	61.3
Ketoconazole	CYP3A	172.9 ± 8.6	25.9	89.6 ± 9.4	1.9	20.0 ± 1.6	77.2	74.3 ± 9.3	6.7

**Table 6:** Effect of CYP450s inhibitors on four lignans metabolism in human liver microsomes.

**Groups**	**CYP450s**	**Schisandrin**		**Schisantherin A**		**Deoxyshisandrin**		γ**-Schisandrin**	
		**Metabolism rate (nmol/min/mg)**	**Inhibition (%)**	**Metabolism rate (nmol/min/mg)**	**Inhibition (%)**	**Metabolism rate (nmol/min/mg)**	**Inhibition (%)**	**Metabolism rate (nmol/min/mg)**	**Inhibition (%)**
Control	–	263.4 ± 22.5	–	179.0 ± 14.8	–	162.5 ± 15.6	–	194.1 ± 12.9	–
Furafylline	CYP1A2	222.3 ± 18.6	15.6	131.6 ± 17.3	26.5	158.6 ± 15.4	2.4	129.9 ± 17.1	33.1
Ticlopidine	CYP2C19	180.2 ± 17.4	31.6	56.2 ± 6.8	68.6	15.3 ± 1.12	90.6	184.6 ± 20.7	4.9
Sulfaphenazole	CYP2C9	167.5 ± 11.2	36.4	115.6 ± 9.2	35.4	156.9 ± 19.1	3.6	180.9 ± 15.8	6.8
Quindine	CYP2D6	249.7 ± 33.1	5.2	172.0 ± 12.3	3.9	111.3 ± 9.2	31.5	172.0 ± 9.7	11.4
DETC	CYP2E1	183.1 ± 16.9	30.5	88.4 ± 6.6	50.6	86.5 ± 8.9	46.8	101.9 ± 5.4	47.5
Ketoconazole	CYP3A4/5	176.7 ± 27.6	32.9	160.4 ± 21.7	10.4	38.2 ± 4.3	76.5	184.0 ± 14.7	5.2

### Transport studies of four lignans across Caco-2 cell monolayer

The transport across Caco-2 cell monolayer was studied to observe the role of multi-drug resistance (MDR) proteins on the *in vivo* disposition process of the four lignans. The results (Table 7) showed that there was no obvious dose-response relationship between the permeability coefficients P_app(AP → BL)_ or P_app (BL → AP)_ and the escalating concentrations of the four lignans (5, 10, and 50 μm). According the values of P_app_ and ER, the four lignans could be classified as moderately absorbed compounds, but no substrates of P-glycoprotein (P-gp), although there was some efflux trend in transport of deoxyschizandrin. The results of digoxin (P-gp substrate), atenolol (poor permeability) and propranolol (high permeability) in Caco-2 cell were consistent with the previously reported data, indicating good reliability and integrity of the Caco-2 cell monolayer model in our study.

**Table 7:** The transport of the four schisandra lignans across Caco-2 cells monolayer model.

**Compounds**		***P_*app*(*AP*−*BL*)_* (× 10^−6^ cm/s)**	***P_*app*(*BL*−*AP*)_* (× 10^−6^ cm/s)**	**ER**
Schisandrin	5 μM	2.15 ± 0.56	3.31 ± 0.59	1.54
	10 μM	1.86 ± 0.17	2.03 ± 0.11	1.09
	50 μM	1.93 ± 0.20	2.12 ± 0.17	1.10
Schisantherin A	5 μM	1.33 ± 0.12	1.52 ± 0.26	1.14
	10 μM	1.81 ± 0.35	2.28 ± 0.47	1.26
	50 μM	1.43 ± 0.35	1.28 ± 0.24	0.89
Deoxyschizandrin	5 μM	1.17 ± 0.13	1.67 ± 0.14	1.42
	10 μM	1.16 ± 0.04	1.52 ± 0.16	1.31
	50 μM	1.24 ± 0.17	1.71 ± 0.51	1.38
γ-Schisandrin	5 μM	1.17 ± 0.16	1.32 ± 0.14	1.13
	10 μM	1.18 ± 0.09	1.34 ± 0.11	1.14
	50 μM	1.22 ± 0.16	1.32 ± 0.20	1.08
Digoxin (5 μM)		3.29 ± 4.02	11.61 ± 0.82	3.53
Digoxin (5 μM)+PSC833		1.46 ± 0.24	2.38 ± 0.58	1.63
Atenolol (100 μM)		0.25 ± 0.03	0.21 ± 0.03	0.84
Propranolol (5 μM)		32.90 ± 6.03	24.62 ± 1.80	0.75

## Discussion

Herbal medicines have been used for treatments of many kinds of diseases for a long time around the world. A general misconception prevails among the general public that herbal medicines are safe and free from adverse effects because of the natural origin. Moreover, most users of herbal products often self-administered along with therapeutic drugs without informing their allopathic doctors (Putthapiban et al., 2017). Actually, “all natural” herbal products are mixtures of potentially biologically active ingredients without clear quantities. These ingredients may possess inherent pharmacological activities, cause unexpected herb-drug interactions and even have direct toxicities (Baig et al., 2014; Banumathi et al., 2017). Meanwhile, it is known that expressions of proteins, activities of enzymes and functions of organs would be altered in pathological conditions. These alternations might exert an important influence on the *in vivo* pharmacokinetics of drugs, and furthermore, affect the therapy, or safety (Yokogawa et al., 2004; Tan et al., 2009). Therefore, it is important and meaningful to reveal the pharmacokinetics of herbal ingredients in pathological conditions for safe and rational application of herbal drugs in clinic.

Most of the previous studies reported that the SC active lignans had hepatoprotective effect against many kinds of liver diseases. However, it was recently revealed that schisandrin B and schisandrae fructus oil could induce hepatotoxicity associated with elevations in hepatic and serum triglyceride levels in mice (Zhang et al., 2014). In addition, it was found that SC lignans exhibited inhibition or induction effects on CYP450s, the most important drug metabolism enzymes, causing herbal-drug interaction which might affect the drug therapy or safety (Wang et al., 2014). Therefore, we conducted the pharmacokinetic study of SC effective components under pathological condition, especially considering the common use of SC preparations for different liver diseases and the key role of liver in disposition of xenobiotics.

CCl_4_ is an often used toxicant for establishment of Acute Liver Injury (ALI) animal model. This agent could be transformed into reactive toxic metabolites by the CYP450 system to cause liver injury, characterized by the elevation of serum ALT and AST, and morphological changes including hepatocyte inflammation, necrosis and steatosis, etc. (Li et al., 2014; Abdel-Bakky et al., 2015; Liang et al., 2015). In our present study, the serum ALT and AST increased by 8.2 and 1.3-folds, respectively. Moreover, BUN, an index of kidney function, was mildly increased in CCl_4_-intoxicated rats. These results was consistent with the data published, suggesting successful establishment of ALI rat model. However, the short-term (4 day) SC treatment to ALI rat did not show hepatotherapeutic effect on liver injury, which was different from the results of SC pretreatment before CCl_4_ injection to rats or long-term application of SC extract to mice (Xie et al., 2010; Cheng et al., 2013; Chen et al., 2017). Meanwhile, after dosing of SC extract to ALI rat, it was found that the phamacokinetic profiles of the four schisandra lignans were obviously different from those in control group, exhibited by elevated Cmax and AUC, prolonged MRT and decreased clearance, all of which might increase the possibility of adverse effects or safety concern. To elucidate the mechanism of the pharmacokinetic alternation of the four lignans in ALI rats, the *in situ* intestinal and hepatic perfusion models were applied for investigation of the respective contributions of intestinal absorption and hepatic disposition. The results suggested that the obviously reduced metabolism of the four lignans in intestine or liver might the main reason for the alternation of the pharmacokinetic profiles.

It has been known that liver CYP450s were main enzymes involved in the *in vivo* metabolism of xenobiotics, including drugs. Therefore, it was very possible that the change in the metabolism of four active lignans was associated with CYP450s. It was reported that the mRNA expressions of liver CYP450s, including CYP2C11, CYP2E1, CYP3A2, and CYP1A2, were depressed in ALI model rat, indicating the reduction of the protein expression and function of liver CYP450 enzymes (Jia et al., 2007). Hence, the activities of liver CYP450s in ALI rat were further determined in our study. The results revealed that the content of liver CYP450 was decreased by 54% in CCl_4_-treated rats, while the activities of six major CYP450s isozymes were also depressed significantly. Similarly, the activities of liver CYP450s from AHC patient were also significantly reduced. Moreover, the *in vitro* metabolism of four schisandra lignans was markly decreased in liver microsomes from CCl_4_-treated rat and AHC patient. The decreased activity of the cytochrome P450 enzymes indicated by the change in the isozyme spectrum after CCL_4_-intoxication was basically coincident with the reduced metabolism of the four lignans in liver microsomes following the application of selective inhibitors of CYP450s. Multiple CYP450s, including CYP1A, CYP2C, CYP2D, CYP2E, and CYP3A, were proved to involve the metabolism of the four schisandra lignans in rat and human liver microsomes. Of course, it was better to confirm the results using recombinant human CYP450 isozymes while the inhibition of CYP450s by specific drug inhibitors only gave a relative answer. It was also observed that four lignans were not the substrates of the drug resistant protein P-gp, suggesting little possibility of involvement of P-gp into the disposition of the four active lignans in CCl_4_-treated rat. Based on all the data in our study, it could be concluded that the depression of the liver CYP450s activities in ALI rat resulted in the pharmacokinetic alternation of the four SC active lignans. When SC preparations were used in clinic, it is better to adjust the dosage according to the liver function and CYP450s activities of patients to avoid toxicity or adverse effects, considering decreased metabolism of the active SC lignans in liver microsomes from AHC patients.

In summary, the pharmacokinetics of four active SC lignans after oral administration was significantly changed when its hepatoprotective effect was not obviously observed after a short-term treatment of SC alcoholic extract to ALI model rat intoxicated with CCl_4_. The decreased metabolism of the lignans in liver or intestine due to the activity reduction of multiple CYP450s involved was believed to contribute the most to the increased exposure of the four SC components in ALI rats. The pharmacokinetic study of the effective SC lignans under the pathological condition of liver dysfunction provided reliable experimental data for rational and safe clinical application of SC preparations as well as the related pharmacological or toxicological research.

## Author contributions

RW: Performed the experiments, analyzed the data, and wrote the manuscript; ZX, XZ, and FL: Performed concentration detection; WZ and YZ: Participated in research design and amended the paper.

### Conflict of interest statement

The authors declare that the research was conducted in the absence of any commercial or financial relationships that could be construed as a potential conflict of interest.
